# The prevalence of elongated styloid process in the population of Barcelona: a cross-sectional study & review of literature

**DOI:** 10.1186/s12903-023-03405-0

**Published:** 2023-09-19

**Authors:** Hassan Assiri Ahmed, Albert Estrugo-Devesa, Xavier Roselló Llabrés, Sonia Egido-Moreno, José López-López

**Affiliations:** 1 Odontological Hospital University of Barcelona, Faculty of Medicine and Health Sciences (Dentistry), University of Barcelona, L’Hospitalet de Llobregat, Spain; 2 Departamento de Odontoestomatología, Pabellón de Gobierno, Campus Universitario de Bellvitge – Universidad de Barcelona, C/ Feixa Llarga s/n, L’Hospitalet de Llobregat, Barcelona, 08907 Spain

**Keywords:** Styloid process, Elongated styloid process, Eagle’s syndrome, Panoramic radiography

## Abstract

**Background:**

Styloid process (SP) is a cylindrical bony projection that originates from the inferior part of the petrous temporal bone just anteriorly to the stylomastoid foramen. Several nerves, muscles, and ligaments are related closely to the (SP). It is considered elongated when the measurement exceeds 30 mm. The overall prevalence of the styloid process is between 3.3% to 84.4%. The elongation of the styloid process (ESP) is associated with the manifestation of Eagle’s Syndrome (ES) which is characterized by various types of pain in the head and neck region such as headache, tinnitus, otalgia, and trigeminal neuralgia. Eagle’s syndrome occurs in 4–10.3% of individuals with an elongated styloid process (ESP). The objective of the study is to determine the prevalence of (ESP) in the patients who were treated in the Dental Hospital University of Barcelona (HOUB), to review the literature to spot the light on the different demographic data worldwide.

**Methods:**

The archived panoramic image in the University of Barcelona dental Hospital were consecutively retrieved to investigate the prevalence of (ESP). Of all digital panoramic radiographs (OPG), 400 met the inclusion criteria and were furtherly analyzed. The results are correlated with the participant’s gender, age, and occurrence. Age is subcategorized into three groups. A chi-square test is used to measure the significant differences and the *P*-value is set at < 0.05 for the level of significance.

**Results:**

Among the included 400, we found 291 demonstrating (ESP). The prevalence of (ESP) which exceeds 30 mm is 72.75%. It is found that the most common morphological type is type 1 which is regarded as the uninterrupted (ESP) regardless of gender and age group. Concerning the calcification pattern, the most prevalent is the partial calcified (ESP) despite genders and age groups.

**Conclusion:**

**(**OPG) is a sufficient tool for the screening of the elongated styloid process. Regarding the prevalence, our results are considered higher than previously reported prevalence in different populations using (OPG) radiography tool. A study on a wider spectrum of the Spanish population is recommended to further investigate the correlation between the elongated styloid process and the occurrence of Eagle’s syndrome.

## Background

The styloid process SP is a cylindrical bony projection that originates from the inferior part of the petrous temporal bone just anteriorly to the stylomastoid foramen [1]. The ‘styloid’ is a term derived from the Greek word ‘Stylos’ which refers to pillar [2]. Embryologically, this apophysis develops from the Reichert’s cartilage of the second pharyngeal arch [3]. It is subjected to ossification from the third trimester of pregnancy through the first ten years of life. Regarding its location, it is positioned laterally in the neck between the internal and external carotid arteries and the internal jugular vein. Several nerves including the glossopharyngeal, facial, accessory, hypoglossal, and vagus are located near the SP [4]. Regarding anatomic structure, the distal part of the styloid process is considered the origin of various muscles including the stylohyoid, stylopharyngeus, and styloglossus. In addition, the stylomandibular ligament and the stylohyoid ligaments emerge from part of the styloid process and are inserted into the ramus of the mandible and the lesser horn of the hyoid bone respectively [5]. The muscles attached to the SP are known as Riolan’s bouquet [6]. He has described the muscle of the styloglossus, stylopharyngeus, and stylohyoid as red flowers whereas the stylomandibular and stylohyoid ligaments as white flowers [6]. These structures contribute to the movement of the oropharyngeal complex. Considering the function of Ronal’s bouquet, the styloglossus is an extrinsic muscle of the tongue in addition to the genioglossus, hyoglossus, and palatoglossus. Styloglossus contributes to the retraction and side elevation movement of the tongue. On the other hand, the stylohyoid muscle acts to elevate the hyoid bone during the process of swallowing. The stylopharyngeus muscle elevates and widens the pharynx during swallowing besides larynx elevation [5]. Concerning the function of the ligaments, the stylohyoid ligament connects the SP to the hyoid bone and acts to elevate it. The stylomandibular ligament is attached to the medial side of the mandible and functions to limit the maximum opening and protrusion of the mandible [7]. ESP is defined as the condition at which the SP exceeds 30 mm when measured from the emergency point in the temporal bone down to the tip of the process [2, 8, 9]. Regarding the prevalence of elongated styloid process ESP*,* several studies have investigated the elongation of the styloid process in different populations. The prevalence ranges between 3.3 to 84.4% (Table 1) [2, 3, 8–43].

**Table 1:** Demographic data concerning the prevalence of elongated styloid process using OPG

**Study**	**Year**	**Demographic population**	**Participants**	**ESP > 30 mm**
Al Zarea et al. [2]	2017	Saudi Arabian	198	44%
Vieira et al. [3]	2015	Brazilian	736	43.89%
Kaufman et al. [8]	1970	American	484	28%
Goldstein & Scopp [10]	1973	American	554	22.2%
Gossman & Tarsitano [11]	1977	American	4200	4%
Correl et al. [12]	1979	American	1771	18.2%
O’Carroll et al. [13]	1984	American	479	35.3%
Monsour and Young et al. [14]	1986	Australian	1200	21.1%
Keur et al. [15]	1986	Australian	1135	30%
Ferrario et al. [16]	1990	Italian	286	84.4%
Bozkir et al. [17]	1999	Turkish	200	4%
MacDonald-Jankowski et al. [18]	2001	Londoners & Chinese (HK)	1662	7.8–8.6%%
Scaf et al. [19]	2003	Brazilian	166	12.6%
Kursoglu et al. [20]	2005	Turkish	55	83.6%
Ilguy et al. [9]	2005	Turkish	860	3.7%
Radfar et al. [21]	2008	Iranian	1000	22%
Gokce et al. [22]	2008	Turkish	698	7.7%
Balcioglu et al. [23]	2009	Turkish	227	3.3%
More & Asrani et al. [24]	2010	Indian	500	19.4%
Öztaş et al. [25]	2012	Turkish	2000	67.5%
Bagga et al. [26]	2012	Indian	2706	52.1%
Shaik et al. [28]	2013	Saudi Arabian	1162	63.2% M; 36.8%F
Alpoz et al. [29]	2014	Turkish	1600	28.8%
Chabikuli et al. [30]	2016	South African	147	69%
Sakhdari et al. [31]	2017	Iranian	500	17.7%
Rai et al. [32]	2017	West Indian	987	27.3%
Gracco et al. [33]	2017	Italian	600	31%
Mathew et al. [34]	2017	Indian (Kerala)	100	35%
Hettiarachchi et al. [35]	2019	Sri Lankan	100	29%
Zokaris et al. [36]	2019	Greek	805	30%
Sharma et al. [37]	2019	Nepalese	1061	57.5% R; 42.3% L
Sridevi et al. [38]	2019	Indian	500	55.8%
Asutai et al. [39]	2019	Turkish (East Aegean)	3678	7.01%
Aoun et al. [40]	2020	Lebanese	489	15.5%
AlSweed et al. [41]	2021	Saudi Arabian	2010	21%
Swapna et al. [42]	2021	Saudi Arabian	300	27.3%
Chen et al. [43]	2022	Taiwanese	593	41.5% R; 36.2% L
Roopashri et al. [44]	2012	Indian	300	35.6%

From the radiographic point of view, there have been varieties in the methods used for the evaluation of the styloid process elongation that ranges from orthopantomography OPG to the most advanced technology of cone beam computed tomography (CBCT) and computed tomography (CT) [45–47]. Nonetheless, OPG is an extraoral imaging technique that depicts the jaws and the surrounding structures in a two-dimensional view. Due to its cost-effectiveness and ease of use, it is considered by most practitioners as the first line of radiographic diagnosis modality for the dentomaxillofacial complex [48–52]. It provides a general perspective on pathological change in the maxilla and mandible. Therefore, OPG aids to distinguish abnormal changes and observe normality. In addition, the state of teeth impaction, dental anomalies, incidental findings, malocclusions, and abnormalities of the structures surrounding the jaws are illustrated using OPG [53]. Consequently, the elongation of the styloid process unilaterally or bilaterally can be addressed via the orthopantomography technique. The elongation of the styloid process is potentially linked to the occurrence of craniofacial and cervical pain; a status described by the American Otolaryngologist Watt. W. Eagle and termed as Eagle’s Syndrome [54]. It is characterized by episodes of pharyngeal pain referred to different areas of the cervicofacial area [47]. It is reported in the literature that approximately 4–10.3% of individuals with elongated SP would develop this type of pain manifestation [47]. Although Eagles’ syndrome has no specific behavior or pain symptoms, patients present with dysphagia and general head and neck pain known as cervicalgia due to the pressure on the nerves and muscles contained in the surrounding structures [55]. Other symptoms including tinnitus, otalgia, and trigeminal neuralgia are linked to the presence of elongated SP [55]. Moreover, the elongated SP process could lead to more serious consequences like transient ischemic attack or stroke because of its proximity and compression on the carotid artery [56]. The etiopathogenesis of Eagle’s syndrome is attributed to different theories including congenital elongation of the styloid process and the calcification of the stylohyoid ligament [57]. Furthermore, previous tonsillectomy is linked to some extent to the occurrence of Eagles’ Syndrome [57].

To our knowledge, the status of styloid process elongation in the Spanish population is not yet described. Accordingly, our main objective is to determine the prevalence of the elongated styloid process in the patients treated at the Dental Hospital University of Barcelona HOUB. The secondary objectives are to review the literature to spot the light on the different demographic data worldwide.

## Methods

### Study design

The present study is regarded as a retrospective descriptive cross-sectional of the archived panoramic radiographs available for the patients treated in the Dental Hospital of the University of Barcelona HOUB, Spain between June 2018 to December 2022. The ethical approval for the study was provided by the ethics committee of HOUB and approved under protocol number 32/2022. The radiographic images are obtained through a simple randomization method and retrieved consecutively from the archived images for analysis.

### Sample size calculation

To identify the sample size needed for this study, the following formula is used (z statistic) * (z statistic) * P * (1-P)/ (precision ^2)^ [56] where: i.-P = expected prevalence of condition (obtained from previous research); ii.-Precision = how much variation in prevalence is acceptable = prevalence - least expected prevalence = allowed error (5–10%); iii.-Standard normal variate (alpha error) known as (z value or z statistic) = 1.96 for confidence interval 95%; iv.-Based on a previous sample size of the study conducted by Gracco et al. [33], our calculation would be as following: (1.96)^2^ * 0.27 * (1–0.27)/ (0.05)^2^ = 302.87 = 303. So, the minimum number of patients that we would include in our study is 303 patients. The sample of 640 images was randomly selected from the total number of radiographs according to the simple randomization method. SP length is measured following Ilgüy et al. [9] the calcification pattern, and morphology types are recorded following Langlais et al. [58]. Additionally, the hospital records of the patients demonstrating ESP were reviewed to identify the possible symptoms matching the ES manifestations including cervicalgia, dysphagia, otitis, migraine, unexplainable chronic pain, and facial muscular pain [59].

**Fig. 1:**
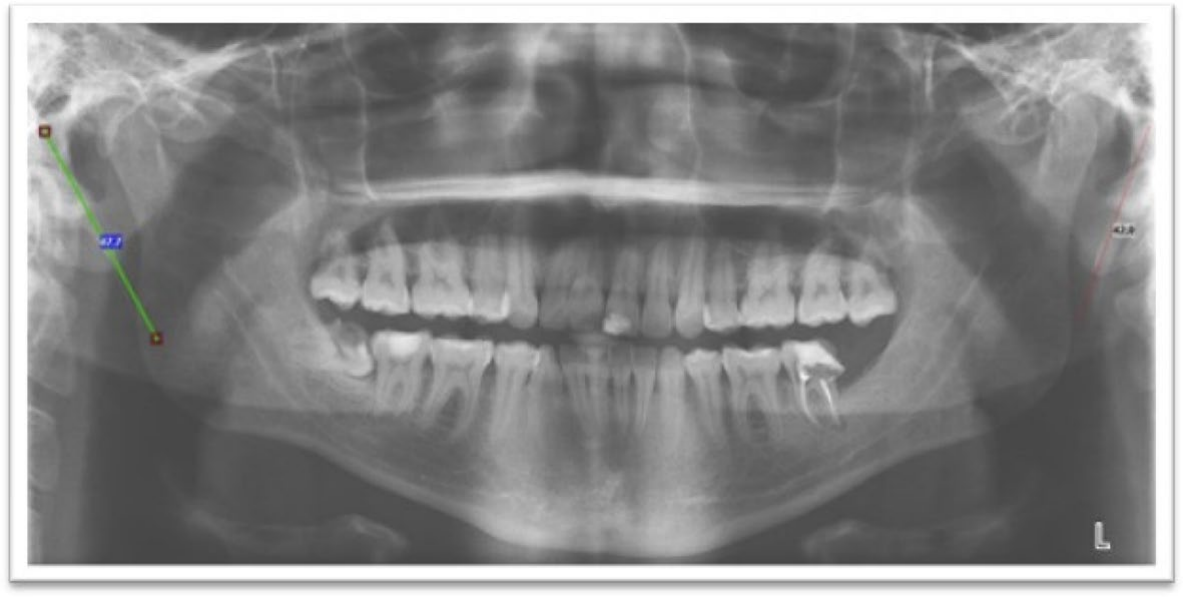
Radiographic illustration of bilateral ESP on digital panoramic radiography with measurements using the Romexis software tool. Image from HOUB

*The inclusion and exclusion criteria in our study were set as follows: *The inclusion criteria include i.- panoramic image free of distortion and errors and ii.- panoramic radiographs for patients > 18 years old. Concerning exclusion criteria, radiographic images that have errors, distortion, or cuts are excluded. Regarding the quality of analyzed images, only images that depicts both styloid processes with adequate quality are considered. OPG images were acquired using a Planmeca ProMax® x-ray unit manufactured by (Planmeca Oy, 00880 Helsinki, Finland) equipped with a digital sensor Planmeca Dimax 3. The measurement tool is performed by the software Planmeca Romexis®. The images were taken based on the manufacturer’s recommendations about the standards of exposure. The Kilovoltage value kV ranges from 64–70 kV and the milliamperage value mA ranges from 7–14 mA based on whether the patient is an adult female, small male, adult male, or large adult male. Well-trained oral radiology doctoral students conducted the investigation twice at the one-month interval and the intra-examiner reliability was calculated using *the Kappa* test. An example of the panoramic image depicting the bilateral elongation of the styloid process (Fig. 1).

### Statistical analysis

For analysis, the data obtained were entered into the excel package 2022. Results and the correlations between genders, age groups, side of elongations, and elongation in mm were analyzed using the Statistical Package for Social Sciences SPSS version 22. In this cross-sectional study, we have applied the Chi-square test to measure the significant differences, and *p*- the value is set at < 0.05 for the level of significance. *The Kappa* test is calculated for intra-observer agreement within one-month intervals [60].

## Results

**Fig. 2:**
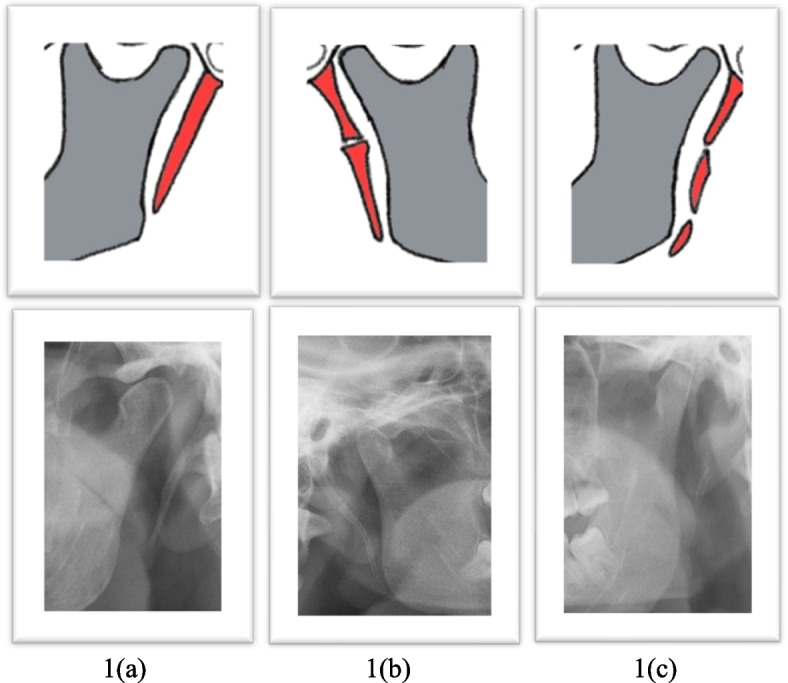
Diagram & radiographic illustration of morphologic types of ESP. **a** Uniterrupted. **b** Pseudo-articulated. **c** Segmented. Drawings by the author HA, images from HOUB

The panoramic radiographs of the patients treated in the dental hospital of the University of Barcelona from June 2018 up to December 2022 were collected. A total of 12,000 panoramic radiographic images were retrieved consecutively from the archived data in the dental hospital. A sample of 640 radiographies are randomly selected from the total number of radiographs according to the simple randomization method, and only 400 met the inclusion criteria while 120 are found to be for patients under 18 years old. Accordingly, there are no data for participants under 16 years old in our study. In addition, about 80 radiographies are disregarded as the radiographic images are distorted whereas 10 images were excluded as the patients fail to provide consent to perform the analysis. Similarly, about 30 radiographies were disregarded due to unclear observation of the styloid process. Accordingly, all 400 radiographies are screened completely without any missing data or images. Measurements of the lengths of styloid processes were conducted on diagnostically acceptable images [58]. According to Ilguay et al. [9], we measured the elongated styloid process starting from the point of origin in the tympanic plate up to the tip of the process using the measuring tool in OPG. SP is considered elongated when it is equal to, or it exceeds 30 mm [9]. In addition, the morphology of the elongated processes was evaluated based on Langlais’ classification as a type of 1 = uninterrupted, type 2 = pseudo-articulated, and type 3 = segmented [58] (Fig. 2).

**Fig. 3:**
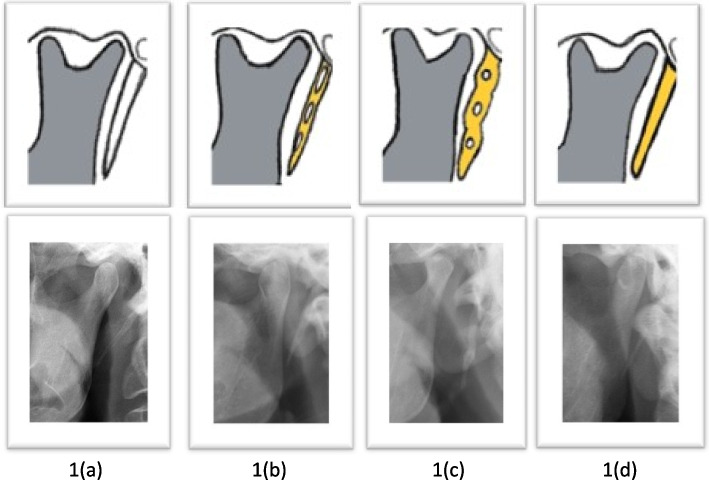
Illustration of ESP calcifications. **a** Outline calcification. **b** Partially. **c** Nodular. **d** Completely calcified. Drawings by the author HA, images from HOUB

The calcification pattern was described and regarded as type A = calcified outlines, type B = partially calcified, Type C = nodular, type D = completely calcified according to Langlais et al. [59] (Fig. 3).

Among the included 400 images, 291 have elongated styloid process either bilaterally or unilaterally which represents 72.75% while only 109 (27.25%) of the investigated radiographies do not show any evidence of ESP. The calculated intra-observer agreement was 0.54 which indicated a moderate level of agreement using the *Kappa* test.

Our results revealed that 72.57% of the participants have ESP compared to 27% with non-ESP, and the difference is statistically significant with *a p*-value of 0.0001 (Table 2).

**Table 2:** Genders number and frequency in association with (ESP) versus (non-ESP)

**Data**	**Males**	**Females**	**Patients with ESP**	**Patients with non-ESP**	**Chi**^**2**^	***P*-value**
*Number*	127	164	291	109	161.8	0.00000001*
*Frequency*	43.64%	56.36%	72.75%	27.25%		

^*^Statistically significant difference

We noticed ESP is more prevalent in females compared to males. Also, regarding age, our data indicate that there is no clinically significant difference in elongation among the different age groups (Table 3).

**Table 3:** Gender & age groups related distribution

**Participants**		**Non- ESP**	**ESP**	**Chi**^**2**^	***P*-value**
*Gender*					
Females	N	67	164	0.849	0.366
	%	29.0%	71.0%		
Males	N	42	127		
	%	24.9%	75.1%		
*Age groups*					
18–30	N	42	84	3.86	0.145
	%	33.3%	66.7%		
31–60	N	46	151		
	%	23.4%	76.6%		
> 60	N	21	56		
	%	27.3%	72.7%		

For the occurrence, the ESP occurs more bilaterally, and the difference is a statistically significant *p*-value of 0.0001 (Table 4).

**Table 4:** Occurrence of unilateral and bilateral (ESP)

**Occurrence**		**Non-ESP**	**ESP**	**Total**	**Chi**^**2**^	***P*-value**
*Non*	N	109	0	109	360.2	0.00000001*
	%	100%	0%	100.0%		
*Unilateral*	N	3	74	77		
	%	3.9%	96.1%	100.0%		
*Bilateral*	N	1	213	214		
	%	0.5%	99.5%	100.0%		
*Total*	N	109	291	400		
	%	27.3%	72.8%	100.0%		

^*^Statistically significant difference

Regarding the calcification pattern, the results signify that the partial calcification type occurs more in females than males with a statically significant difference *p*-value of 0.000. In addition, it occurs more in the age group 31–60 with a statistically significant difference of *p*-value 0.000. Likewise, Type B is more frequent on the right side than the left side with a statistically significant difference *p*-value of 0.000 (Table 5).

**Table 5:** ESP concerning calcification pattern

**Participants**	**Calcification pattern**				**ESP (N)**	**Chi**^**2**^	***P*-value**
	**Type A** **(N)** **(%)**	**Type B** **(N)** **(%)**	**Type C** **(N)** **(%)**	**Type D** **(N)** **(%)**			
*Gender*							
Male	(54) (23.58%)	(84) (36.68%)	(50) (21.83%)	(41) (17.90%)	229	540	0.000*
Female	(31) (11.27%)	(127) (46.18%)	(45) (16.36%)	(72) (26.18%)	275		
*Age group*							
18–30	(14) (9.33%)	(77) (51.33%)	(30) (20%)	(29) (19.33%)	150	22.61	0.000*
31–60	(44) (17.60%)	(101) (40.4%)	(44) (17.60%)	(61) (24.40%)	250		
> 60	(27) (28.42%)	(25) (26.31%)	(21) (22.10%)	(22) (23.15%)	95		
*Side of elongation*							
Right	(42) (16.15%)	(118) (45.38%)	(52) (20%)	(50) (19.23%)	260	514	0.000*
Left	(45) (18.44%)	(97) (39.75)	(46) (18.85%)	(64) (26.22%)	244		

^*^Statistically significant difference

Moreover, our data reveal that morphological type 1 is more prevalent in males. In addition, it is more frequent in the age group 31–60 with a statistically significant difference of *p*-value 0.0001. This type is also more prevalent on the right side compared to the left side (Table 6).

**Table 6:** ESP concerning the morphology

**Participants**	**Morphology**			**ESP** **(N)**	**Chi**^**2**^	***P*-value**
	**Type 1** **(N)** **(%)**	**Type 2** **(N)** **(%)**	**Type 3** **(N)** **(%)**			
*Gender*						
Male	(116) (50.65%)	(45) (19.65%)	(68) (29.69%)	(229)	3.15	0.187
Female	(136) (49.45%)	(57) (20.72%)	(82) (29.81%)	(275)		
*Age groups*						
18–30	(68) (45.33%)	(24) (16%)	(58) (38.66%)	(150)	35.3	0.0001*
31–60	(108) (43.2%)	(62) (24.8%)	(80) (32%)	(250)		
> 60	(710 (74.7%)	(13) (13.68%)	(11) (11.75%)	(95)		
*Side of elongations*						
Right	(123) (47.30%)	(55) (21.15%)	(82) (31.5%)	(260)	1.15	0.562
Left	(127) (52.04%)	(48) (19.67%)	(69) (28.27%)	(244)		

^*^Statistically significant difference

On the other hand, we noticed that many patients demonstrated symptomatic ESP. These patients represent approximately 9.6% of all patients with ESP. The symptoms are more prevalent in females 64.28% compared to males 35.71%. Statistically, there is no significant difference in gender. Regarding age, the symptoms are more frequent in the group aged 31–60 years old. However, there are no statistically significant differences between the age groups.

## Discussions

Generally, the styloid process is considered one of the incidental findings that are encountered in patients during routine dental examinations using panoramic radiography. In our study, we performed the measurements and investigations on digital OPG. This diagnostic technique is affordable and can be easily performed and interpreted [61]. Aside from exposing patients to a lower radiation dose, this method is much more cost-effective than CT [42]. Since digital panoramic radiography is commonly prescribed in dental practice including a styloid process elongation analysis. We decided to use this diagnostic modality in the present study. For the prevalence of the elongated styloid process in our sample, we found that it manifests in 72.5% of the study’s participants. This agrees with the results reported by Ferrario et al. [16] and Kursoglu et al. [20] whom they regarded prevalence as more than 70% of their populations. Concerning gender distribution of the elongated SP, our study postulates that the elongation is more in females 164 (56.36%) than in males 127 (43.64%). It is following the study published by Omami et al. [62], and Vieira et al. [3], Ferrario et al. [16], Roopshari et al. [44]. Additionally, the estimated difference between females and males is not clinically significant in the studies of Ferrario et al. [16] and Roopashri et al. [44]. Our findings regarding gender predilection disagree with those reported by AlZarea et al. [2], Rai et al. [32], and Sokler et al. [63] who declared more prevalence in favor of males. Statistically, our results are following the study conducted by Gokce et al. [22] on the Turkish population which indicates no clinical significance of elongation between males and females. Regarding the occurrence, our sample reveals that bilateral elongation is the most frequently noted in males and females of all age groups. This is contradictory to the findings reported by Hettiarachchi et al. [35] in which they indicated 26% unilateral elongation and only 11% of bilateral elongation of their sample. On the other hand, it agrees with the studies documented by Sakhdari et al. [31], Vieira et al. [3], and Alsweed et al. [41] for which they postulated more occurrence of bilateral elongation.

Regarding the occurrence, we found that bilateral ESP is more prevalent than unilateral ESP. Our data agree with Ferrario et al. [16], and Zaki et al. [64]. Our study is also following Gosh and Dubey et al. [65] whom they reported bilateral elongation in about 57% of their sample. Similarly, our findings are following Zokaris et al. [36] whom they reported more prevalent bilateral occurrences than unilateral ones. However, they focused their studies on young individuals rather than older ones.

Concerning the calcification pattern described by Langlais et al. [58], our findings indicated that partial calcification type B is the most prevalent among genders of all age groups. Our findings on calcification patterns are different from the other conducted studies on a different population. These studies are those of Shaik et al. [28] and More and Asrani et al. [24], Shah et al. [66] in which they determined that the outline calcification pattern type A is the most prevalent in their population. In addition, our finding is different from the data reported by Khashyab et al. [27] in which they indicated the complete calcification type D is the most common in their sample without any significant difference between the type and pattern in both the right and left sides.

About the distribution of morphology, we noticed that the uninterrupted Type1 is the most common morphological type among all age groups in both genders. This is following More and Asrani et al. [24] who similarly indicated that uninterrupted elongation is more prevalent among other types. Likewise, Shaik et al. [28] reported that uninterrupted elongation is the most prevalent regardless of the side’s occurrence.

Considering the manifestation of Eagle’s syndrome, our data indicated the appearance of the syndrome’s symptoms in women more than men. It consequently agrees with data reported by Keur et al. [15], Liu et al. [67], and Yavuz et al. [68]. Therefore, our results demonstrate a prevalence of 9.6% which is within the worldwide prevalence of 4–10.3%.

We can speculate from the literature that the variation in the elongation of the styloid process as well as morphology and calcification pattern is unclear and could be linked to differences of ethnicity origins or the factors related to the diagnostic method being used. Additionally, the variations in the different studies could be related to the variations in defining a starting point from which the elongation is measured. In our study, we measured the elongation according to Ilgüy et al. [9] which initiated the measurements at a point where the styloid process emerges from the temporal bone. However, some studies measure the length at a point at the line that connects the mastoid to the nasal spine [16, 28, 69].

It seems that the demographic variables of ESP are not consistent. Some studies indicated ESP is proportional to the aging process [29, 70]. Concerning gender, Hamedani et al. [71] indicated that females with low bone density show an ESP as twice as normal ones. Even some studies that were conducted in the same country of India demonstrated different prevalence. Bagga et al. [26] claimed that the higher prevalence in their sample compared to those performed by Phulambrikar et al. [72] and More and Asrani et al. [24] is related to a combination of various factors including lifestyle, race, and dietary intakes. The population is known for chewing hard foods and carrying heavy kinds of stuff on their head that could aggravate the elongation and ossification of ligaments of SP. Another reason for the variability between different studies is related to the method of measurement. Some studies measure the elongation at the point of origin from the temporal bone which is the method we performed and presented similar results of ESP. Other studies consider the measurement at the line that connects the nasal spine to the mastoid process [16, 28, 69].

### Limitations

It would not be ignored that there is possibly some limitation to our study. A larger sample size would provide more information about the estimated prevalence as well as the morphologic and calcification patterns. However, based on the sample size calculation, it is estimated that the minimum number that we could have in our study to reach reliable information is 303 patients.

Indeed, using the most advanced three-dimensional technique; CBCT would provide more details specially the degree of angulation in medial and lateral directions. Above all, this study provides valuable knowledge of the anatomical variations of the status of the styloid process which guides clinicians from different specialties to diagnose Eagle’s syndrome. Yet, the accurate diagnosis of ESP must be assisted by CBCT as it provides details in three-dimensional aspects [73].

In addition, this study is conducted on patients visiting the dental hospital of the university of Barcelona in which the patients are mostly coming from the residential area of Baix Llobregat.

This aspect is a limitation and studies that include a broader Spanish population would be interesting. Though the *Kappa* result agreement indicates a moderate level, the results were interpreted by a well-trained doctoral student who specializes in oral radiology interpretation. The retrospective nature of this work has an intrinsic risk of information and detection bias. It is suggested to emphasize their control in future prospective studies as well as analysis of possible confounding factors and potential differential diagnoses.

## Conclusion

OPG is a sufficient tool for the screening of ESP. Regarding the prevalence, our results are considered higher than most of the previously reported prevalence in different populations using OPG. Mostly, ESP should be considered an asymptomatic entity and should be detected coincidentally during routine dental diagnosis. However, dentists must be aware of cases present with craniofacial pain associated with ESP. Consequently, A study on a wider spectrum, assisted by both OPG and CBCT, of the Spanish population is recommended to further investigate the correlation between the elongated styloid process and the occurrence of Eagle’s syndrome.

## Data Availability

The datasets used and/or analyzed during the current study are available from the corresponding author upon reasonable request.

## Abbreviations

OPG: Orthopantomography
CBCT: Cone beam computed tomography
CT: Computed tomography
ESP: Elongated styloid process
Non-ESP: Non-elongated styloid process
SP: Styloid process
HOUB: University of Barcelona Dental Hospital
kV: Kilovoltage
mA: Milliamperage
SPSS: Statistical package for social sciences
